# Classification of difficult videolaryngoscopic tracheal intubation with different blade types: a prospective external validation study of the VIDIAC score

**DOI:** 10.1111/anae.16678

**Published:** 2025-06-29

**Authors:** Vera Köhl, Viktor A. Wünsch, Thorsten Dohrmann, Linda Krause, Martin Petzoldt

**Affiliations:** ^1^ University Medical Centre Hamburg‐Eppendorf Hamburg Germany

Consistent documentation of videolaryngoscopy findings is pivotal for personalised, targeted airway planning. The videolaryngoscopic intubation and difficult airway classification (VIDIAC) is a prospectively developed and validated universal classification for videolaryngoscopy that shows excellent diagnostic performance and calibration [1] and outperforms the Cormack and Lehane classification in adults and children [1, 2]. It comprises six items: impaired epiglottis movement; increased lifting force; direct epiglottis lifting; vocal cords clearly visible; vocal cords not visible; and enlarged arytenoids (see Fig. 4 and online Supporting Information Figure S2 in [1]), which were identified as the most important factors out of a large set of candidates using a data‐driven approach [1].

The VIDIAC items are interrelated, making it an adoptive score that covers users' personal preferences and decisions. Blade geometry and users proficiency affect device performance substantially [3]. Hyperangulated videolaryngoscopy provides a better glottic view than Macintosh videolaryngoscopy, which translates into faster tracheal intubation and higher success rates when used by experienced airway operators on difficult airways [3].

The VIDIAC score was developed for Macintosh videolaryngoscopy and it remains uncertain if it classifies hyperangulated videolaryngoscopy reliably. This designated secondary analysis of the prospective BLADESHAPE study aimed to externally validate the VIDIAC score separately for Macintosh videolaryngoscopy and hyperangulated videolaryngoscopy and determine if integrating the ‘blade type’ improved its diagnostic performance. We included 182 adults with expected difficult airways who were allocated randomly to either Macintosh or hyperangulated videolaryngoscopy (C‐MAC™, Karl Storz SE & Co KG, Tuttlingen, Germany). Details are reported elsewhere [3].

The primary outcome was the incidence of difficult videolaryngoscopic tracheal intubation (defined as a difficult airway alert documented in patients' electronic health records [1]). We investigated the relationship between the primary outcome and the VIDAC score. Airway operators assessed all VIDIAC predictor variables that were used to calculate the VIDIAC score (range −1 to 5) and primary outcome probability using the ß‐coefficients from the previously reported multivariable regression (VIDIAC) model [1]. We calculated the area under the receiver operating characteristic curve (AUROC) with a 95%CI computed by 2000 stratified bootstrap replicates. Patients were assigned to four groups using the validated severity grading system: ≤ 0 points ‘easy’; 1 point ‘moderate’; 2 points ‘hard’; and ≥ 3 points ‘severe’ videolaryngoscopic tracheal intubation [1]. To evaluate whether integration of the ‘blade type’ in the score might add incremental diagnostic value, we calculated a modified VIDIAC score by adding a value of +1 if hyperangulated videolaryngoscopy was used. Statistical analysis was performed with R version 4.4.2 (R Foundation for Statistical Computing, Vienna, Austria).

Difficult videolaryngoscopic tracheal intubation was recorded in 66 (36%) patients: 43 (47%) in the Macintosh videolaryngoscopy group; and 23 (25%) in the hyperangulated videolaryngoscopy group (Table 1). Impairments regarding the epiglottis‐blade interaction as well as glottic view restriction were observed less frequently with hyperangulated videolaryngoscopy (Fig. 1). The distribution of difficult videolaryngoscopic tracheal intubation rates was balanced between the Macintosh and hyperangulated videolaryngoscopy subsets for corresponding VIDIAC grades (Table 1). The AUROC (95%CI) was 0.93 (0.88–0.97) in the entire study cohort: 0.95 (0.90–1.00) in the Macintosh videolaryngoscopy group; and 0.89 (0.79–0.98) in the hyperangulated videolaryngoscopy group (online supporting information Figure S1). Overall calibration was good (observed/expected ratio 0.96) and the GiViTI calibration belt showed a strong agreement between the observed and predicted probabilities (online Supporting Information Figure S2) [4]. Integration of the ‘blade type’ in the VIDIAC score did not improve its predictive performance (AUROC (95%CI) 0.92 (0.87–0.97) vs. 0.93 (0.88–0.97), p = 0.54) (online Supporting Information Figure S3).

**Table 1:** Incidence of difficult videolaryngoscopic tracheal intubation (primary outcome) in relation to the VIDIAC score. Values are number/total number per category (proportion).

		Overall	HA‐VL	Mac‐VL
		n = 182	n = 91	n = 91
Difficult tracheal intubation		66 (36%)	23 (25%)	43 (47%)
**VIDIAC grade**	**VIDIAC score**	**Difficult tracheal intubation per VIDIAC score**		
Easy to moderate	≤ 1 point	12/127 (9%)	7/74 (9%)	5/53 (9%)
Hard	2 points	16/16 (100%)	8/8 (100%)	8/8 (100%)
Severe	3 points	16/17 (94%)	5/6 (83%)	11/11 (100%)
	4 points	16/16 (100%)	2/2 (100%)	14/14 (100%)
	5 points	6/6 (100%)	1/1 (100%)	5/5 (100%)
**Diagnostic performance of the VIDIAC score to predict the primary outcome**				
AUROC (95%CI)		0.93 (0.88–0.97)	0.89 (0.79–0.98)	0.95 (0.90–1.00)

AUROC, area under the receiver operating characteristic curve; HA‐VL, hyperangulated videolaryngoscopy; Mac‐VL, Macintosh videolaryngoscopy; VIDIAC, videolaryngoscopic intubation and difficult airway classification.

**Figure 1:**
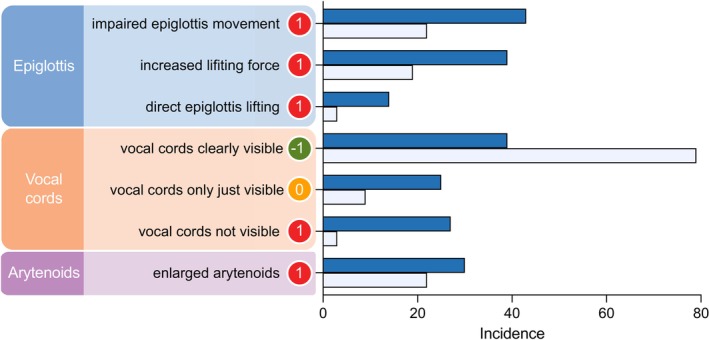
Distribution of videolaryngoscopic intubation and difficult airway classification (VIDIAC) predictor variables in patients in the hyperangulated videolaryngoscopy (light blue bars) and Macintosh videolaryngoscopy (dark blue bars) subsets. VIDIAC score values are given as encircled numbers; x‐axis: incidence of the respective VIDIAC predictor variable as absolute value (n).

This external validation study revealed high diagnostic performance and good calibration of the VIDIAC score for Macintosh and hyperangulated videolaryngoscopy and reproduced the findings from the original VIDIAC study [1], proving its robustness and generalisability. This seems remarkable as our validation cohort [3] differed substantially from the cohort of the original development study [1], not only in terms of blade types, but also regarding users proficiency and adjunct application rules. Interestingly, the incidence of difficult videolaryngoscopic tracheal intubation was lower with hyperangulated videolaryngoscopy (25%) compared with Macintosh videolaryngoscopy (47%); however, as this large difference was reflected adequately by the VIDIAC score ratings, overall distribution of the VIDIAC scores/grades was balanced. External validation is paramount as the clinical application of unvalidated classifications or those with known poor diagnostic performance might lead to unfavourable treatment decisions with the potential of harming patients [5]. Our analysis confirmed that the ‘blade type’ does not need to be integrated in the VIDIAC score.

In conclusion, the VIDIAC score can be applied universally and indiscriminately in daily clinical practice and research to classify videolaryngoscopy with different blade types, owing to its high reproducibility and excellent discrimination.

## Supporting information


**Figure S1.** Receiver operating characteristic curves for the videolaryngoscopic intubation and difficult airway classification (VIDIAC) score for the prediction of difficult videolaryngoscopic tracheal intubation in the entire study cohort, Macintosh videolaryngoscopy and hyperangulated videolaryngoscopy subsets.
**Figure S2.** The GiViTI calibration belt for the videolaryngoscopic intubation and difficult airway classification (VIDIAC) model in the BLADESHAPE cohort outlines the agreement between the observed and expected probabilities of the primary outcome.
**Figure S3.** Receiver operating characteristic curves for the prediction of difficult videolaryngoscopic tracheal intubation for the videolaryngoscopic intubation and difficult airway classification (VIDIAC) score and the modified VIDIAC score that includes the ‘blade type’.
