# Supplementary material for: Classification of difficult videolaryngoscopic tracheal intubation with different blade types: a prospective external validation study of the VIDIAC score
Source: Anaesthesia. 2025 Jun 29;80(9):1137–9. doi: 10.1111/anae.16678 (PMC12351220; doi:10.1111/anae.16678)

**Figure S1** Receiver operating characteristic curves for the VIDIAC score for the prediction of difficult videolaryngoscopic tracheal intubation in the entire study cohort (red line), Macintosh videolaryngoscopy (grey line) and hyperangulated videolaryngoscopy (blue line) subsets.


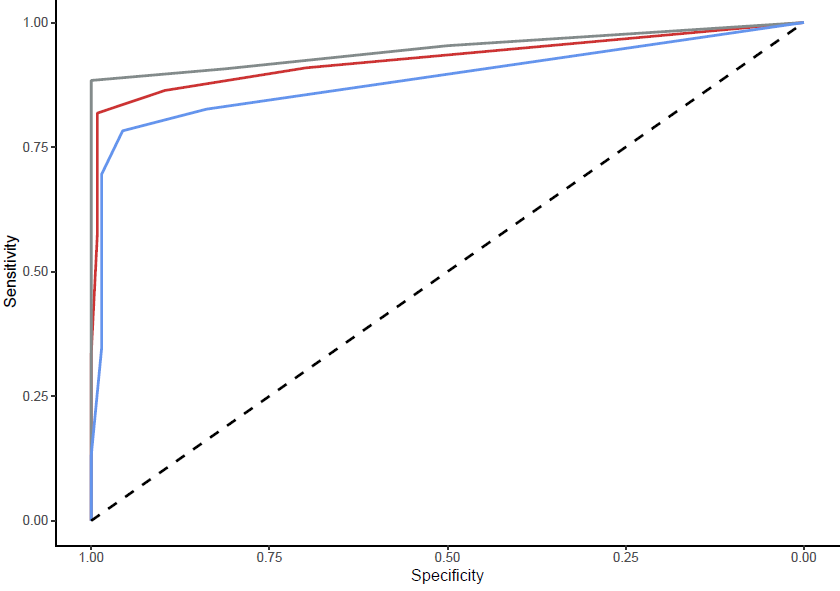


**Figure S2** The GiViTI calibration belt for the VIDIAC model in the BLADESHAPE cohort outlines the agreement between the observed and expected probabilities of the primary outcome. Red line, perfect agreement; dark grey, 80%CIs; light grey, 95%CIs.


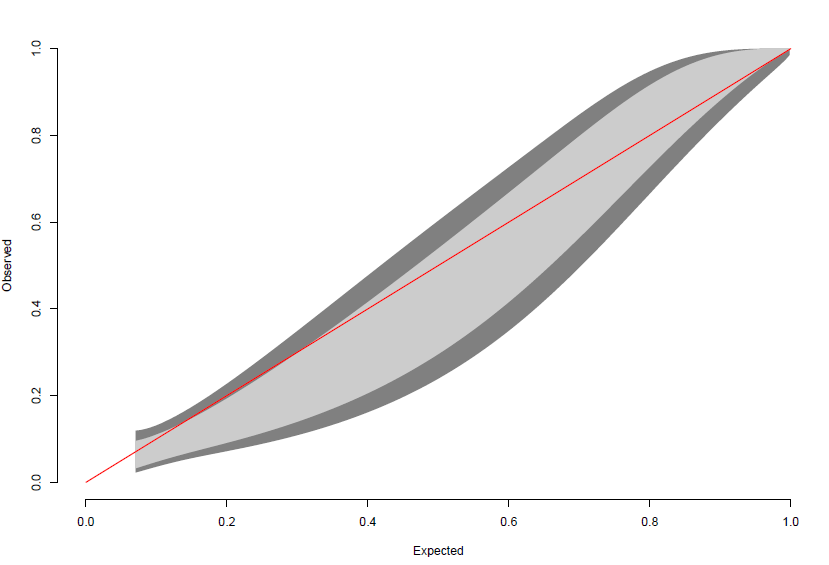


**Figure S3** Receiver operating characteristic curves for the prediction of difficult videolaryngoscopic tracheal intubation for the VIDIAC score (red line) and the modified VIDIAC score, that includes the ‘blade type’ (MAC-VL vs. HA-VL) (rose line)


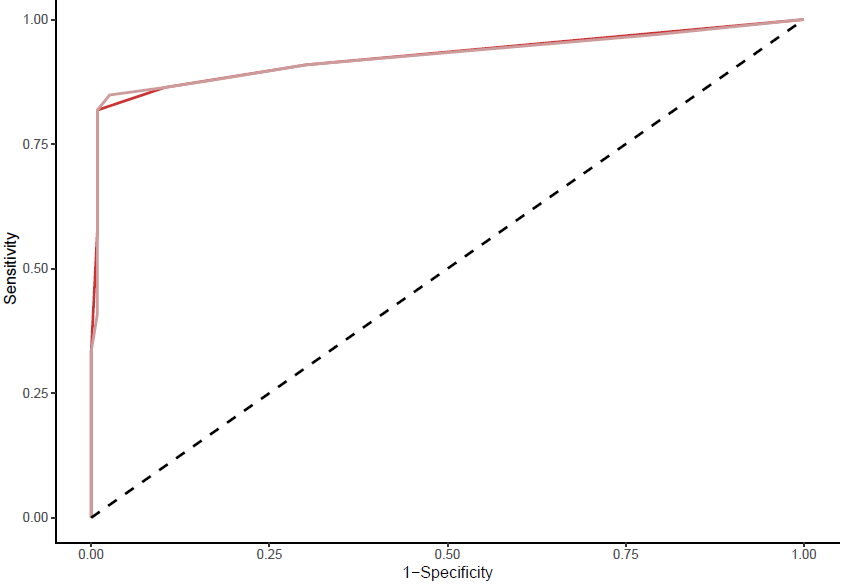

Supplement: Supplementary file 1 — Figure S1. Receiver operating characteristic curves for the videolaryngoscopic intubation and difficult airway classification (VIDIAC) score for the prediction of difficult videolaryngoscopic tracheal intubation in the entire study cohort, Macintosh videolaryngoscopy and hyperangulated videolaryngoscopy subsets. Figure S2. The GiViTI calibration belt for the videolaryngoscopic intubation and difficult airway classification (VIDIAC) model in the BLADESHAPE cohort outlines the agreement between the observed and expected probabilities of the primary outcome. Figure S3. Receiver operating characteristic curves for the prediction of difficult videolaryngoscopic tracheal intubation for the videolaryngoscopic intubation and difficult airway classification (VIDIAC) score and the modified VIDIAC score that includes the ‘blade type’. [file ANAE-80-1137-s001.docx]
